# Management of Bilateral Condylar Fracture With ORIF Using the Puppet Technique in the Adolescence: A Case Report

**DOI:** 10.1155/crid/2285396

**Published:** 2026-08-17

**Authors:** Refitia Inayah Putri, Bramasto Purbo Sejati, Yosaphat Bayu Rosanto

**Affiliations:** ^1^ Resident Oral and Maxillofacial Surgery Study Program, Faculty of Dentistry, Universitas Gadjah Mada, Yogyakarta, Indonesia, ugm.ac.id; ^2^ Oral and Maxillofacial Surgery Study Program, Faculty of Dentistry, Universitas Gadjah Mada, Yogyakarta, Indonesia, ugm.ac.id

**Keywords:** bilateral condylar fracture, facial growth, open reduction internal fixation

## Abstract

**Introduction:**

Condyle fracture may result in functional and cosmetic issues. Malocclusion becomes one of the adverse effects of the fracture that impairs the quality of life. It is crucial for maintaining the mandibular condyle because it is an essential component of jaw movement. The critical decision for the treatment choice in early adolescence has an impact on accurate reduction and rigid fixation. Improper methods of therapy might have major consequences for facial growth and development, with ankylosis as a frequent occurrence.

**Importance:**

To report a case of bilateral condylar fracture in pediatrics and its management and long‐term follow‐up.

**Case Presentation:**

A 16‐year‐old male was referred to the Oral and Maxillofacial Surgery Department after a road traffic accident with complains of pain in the area of TMJ bilaterally. The clinical examination showed an anterior open bite and restricted mandibular movement. CT scan reported a bilateral fracture at the neck condyle, as well as on the right side of the coronoid process, the right side of the symphysis, and the left side angle of the mandible.

**Case Management:**

Open reduction internal fixation (ORIF) and the puppet technique are performed under general anesthesia. Temporary immobilization methods involve arch bars and heavy elastics. Intraoral, submandibular, and miniretromandibular incisions were given concerning the fracture site. A 3‐year follow‐up revealed an occlusion, facial growth, and bone union, which followed the removal of the surgical miniplate.

**Conclusion:**

The patient achieved bone union, occlusal stability, facial development, and recovery of jaw movement on both sides. The treatment also successfully maintains vertical dimensions, which are key to treating such cases.

## 1. Introduction

The face is a structural unit that consists of multiple compartment that plays a critical function in identifying an individual and are responsible for various vital functions. Facial trauma occurs frequently, in consequence of its prominent and superior position. In addition, its bones are fragile and vulnerable to a variety of trauma [1]. Maxillofacial trauma affects multiple structures in the craniofacial skeleton, including soft and bony components of the face, as a result of energy action. In pediatric patients, the most prevalent cause of the fracture is accidental falls (58.2%), including falling from height, followed by violence (12.7%), bicycle (10%), and motor vehicle accidents (8.2%). Sports injuries represented only 7.3%, and others represented 3.6% [1, 2].

The mandible consists of several regions, ranging from the symphysis, parasymphysis, body, ramus, angle, and the condylar and coronoid processes [3]. Mandible fracture accounts for approximately 19%–40% among all facial fractures [4]. At a rate of 25%–35%, condylar fractures rise to the second most common position. Contrary to bilateral fractures, unilateral condylar fractures happen three times more commonly [5]. Bilateral condylar fractures comprise approximately 40%–50% of all condylar fractures [6]. The higher occurrence of mandibular condylar process fractures is due to a large and inflexible mandibular ramus attaching to the condyle via a thin and narrow condylar neck. Condylar fractures can be managed using either conservative or surgical methods [7, 8].

The condylar process is a vital component of the mandible′s development. As a result, any injury to the condylar process in children or teenagers may disrupt growth potential and have long‐term consequences [8]. Occlusal abnormalities, discomfort, masticatory dysfunction, restricted mandibular motions, facial asymmetry, temporomandibular joint problems, or ankylosis are long‐term complications that might reduce quality of life [5]. Facial asymmetry caused by a reduction in the height of the condylar process has frequently been reported [9].

Treatment was difficult due to the anatomical position of the condylar process. The debate over open versus closed treatment of condylar fractures has raged for years. However, a recent meta‐analysis and systematic review have shown that ORIF is beneficial [10]. The quality and quantity of bone at the insertion site have a significant impact on the stability of the miniplates. *This factor contributes to the complexity of managing condylar fractures, primarily due to the limited availability of bone in the region.* Miniplate fixation is a reliable method for providing firm bone anchorage and facilitating healing [11]. Improper methods of bilateral condylar fractures therapy might have major consequences in a variety of abnormalities. This study was designed to prove the efficiency and surgical outcome of bilateral neck condylar fractures in pediatric patients after the use of ORIF approaches and the puppet technique.

## 2. Case Presentation

**Figure 1:**
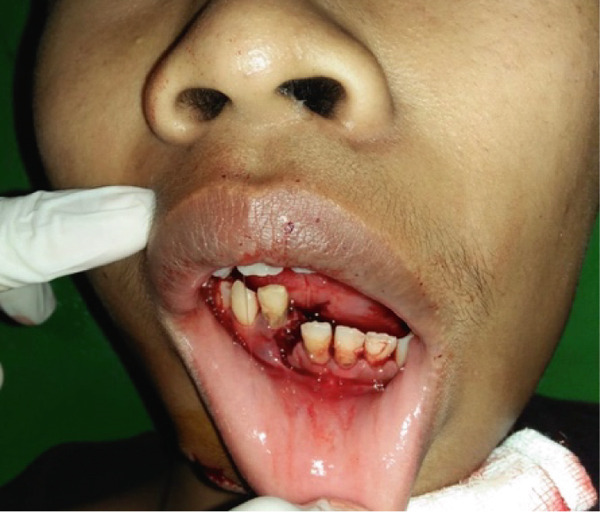
Clinical examination.

This case report was reported according to the CARE guideline. A 16‐year‐old male was referred to the Oral and Maxillofacial Surgery Department of Temanggung Hospital, Central Java, after a road traffic accident. The patient fell and landed on the chin and complained of pain and discomfort in the area of TMJ on the right and left sides, and restricted mouth opening. There was no history of unconsciousness. Antitetanus vaccine and antibiotics were prescribed. The patient had no medical history or allergies. On clinical examination, slight asymmetry was noted over the right and left TMJ areas, associated with tenderness over the TMJ bilaterally. Lacerations were seen over the chin region. Intraoral examination showed deranged occlusion with an anterior open bite, restricted lateral movement of the mandible, and step deformity in the right‐side symphysis region between 41 and 42 teeth. Examination of the patient revealed that mouth opening was restricted to 15 mm on the day of presentation (Figure 1).

**Figure 2:**
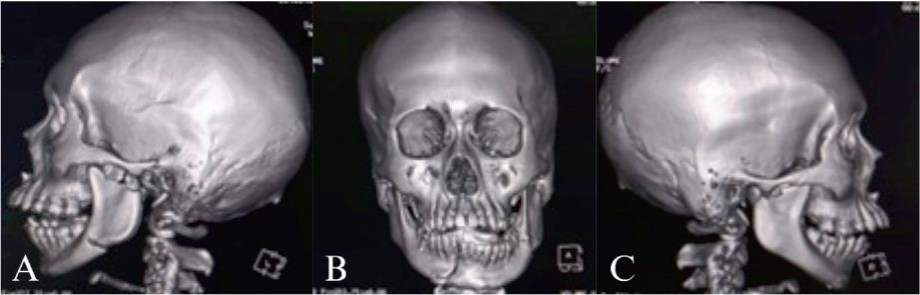
(A) CT showing medially displaced left condylar and angle of mandible fracture. (B) Symphysis fracture line. (C) The right neck condylar fracture.

A computed tomography (CT) scan reported a bilateral fracture at the neck condyle with medial displacement of both the condylar heads. The radiographic examination also showed fracture lines on the right side of the coronoid process, on the right side of the symphysis, and the left side of the mandible (Figure 2). The aim of the therapy for this mandibular fracture case was to achieve occlusion, maintain posterior vertical height, and address functional and esthetic concerns. The patient was diagnosed with bilateral mandibular condylar fracture.

## 3. Case Management

**Figure 3:**
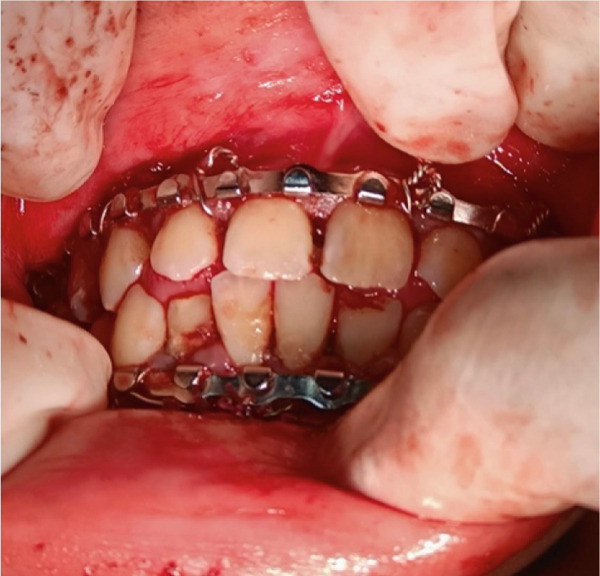
Arch bar splinting.

The surgical procedure was performed under general anesthesia. The nasotracheal intubation was done. Arch bar splinting of the maxilla and mandible was applied. Mandibular vestibular incision was utilized to expose the symphysis fracture line, and the mucoperiosteal flap was reflected. The bridle wire was inserted on both sides of the fracture line to facilitate the fragments′ contact. Bone fixation was done using double 2.0‐mm four‐hole titanium miniplate with 10 and 8‐mm screws (Figure 3).

The submandibular approach was selected to expose the angle mandibular fracture line. The incision was parallel to the inferior border of the mandible for maximum cosmetic benefit. The miniretromandibular skin incision [12] was done 1.5–2 cm below the inferior border of the mandible with a short posterior extension parallel to the posterior border of the mandible. Subcutaneous and underlying platysma were undermined bluntly with scissors before dividing it with a scalpel. The facial nerve and facial blood vessel were identified and ligated. The pterygomassetric sling was incised at the inferior border, and the fracture site was exposed. After reduction, fixation of the fracture segments was done along the external oblique ridge according to Champy′s concept using double 2.0‐mm titanium four‐hole miniplate and 8‐mm screws.

**Figure 4:**
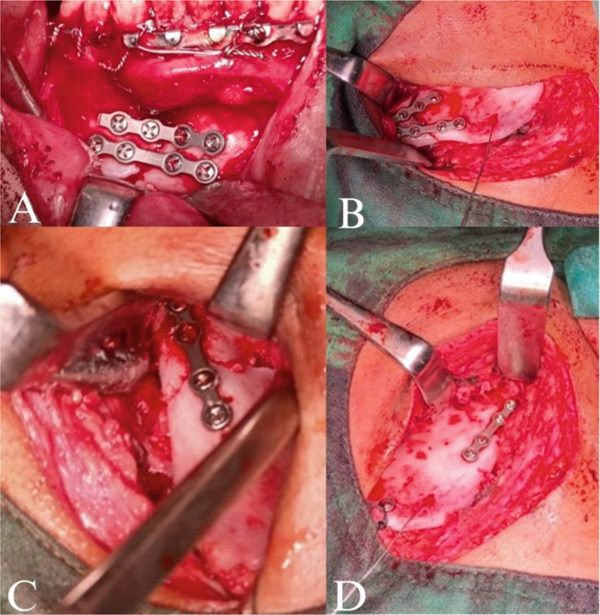
(A) Symphysis fracture. (B) Pull wire for condyle reduction. (C) Intraoperative showing miniretromandibular incision and puppet technique on the right condylar. (D) Left condylar fractured bone.

The blunt dissection was extended in layers to show the left neck condylar fracture. The 0.5‐mm pull wire was inserted in the mandibular angle region by drilling a hole for attachment. The fragment was pulled inferiorly to expose the condylar fracture fragment. Kocher forceps were used to reduce and stabilize the fracture fragment. In this stage, the manipulation of the fracture fragment using Kocher forceps and the mandibular ramus using pull wire mimics a movement of a Javanese Wayang puppeteer, therefore called the puppet technique (Figure 4).

After the reduction was completed, fixation was done using a 2.0‐mm four‐hole straight miniplate and 6‐mm screws. The right side condylar fracture was exposed through the miniretromandibular approach and puppet technique. The right side of the condylar fracture was opened similarly, and reduction and fixation of the fracture were done with a 2.0‐mm four‐holed titanium miniplate. After reduction, bone plating was done, and hemostasis was achieved. The wound was closed in layers to realign the anatomic structure.

The timeline of the treatment is provided in Table 1. Postoperatively, on Day 1, occlusion and jaw movements were checked along with any deficits of facial nerve branches. No abnormalities were noted. The patient was on a soft diet from the first postoperative day and was discharged in good general condition on the third postoperative day. Postoperatively, the patient was given intravenous antibiotics and analgesics for 3 days and oral medications for 5 days at discharge. Local cold application and home oral care instructions were advised. The patient still needs intermaxillary fixation for 2 weeks postoperatively to correct occlusion and deviation. The patient reported that the 2‐week period of intermaxillary fixation was uncomfortable, particularly with regard to speech and oral hygiene maintenance, but was tolerable knowing it was necessary for achieving correct occlusion.

**Table 1:** Summary of the treatment timeline.

Period	Events
Admission day	Road traffic accident—fall onto chin, bilateral TMJ pain, restricted mouth opening (15 mm). Presentation to OMFS Department, Temanggung Hospital—clinical exam, antitetanus/antibiotics, CT scan confirming bilateral condylar neck fractures plus right coronoid, right symphysis, and left mandibular angle fractures
Surgery day	ORIF with puppet technique under general anesthesia (arch bar splinting, miniplate fixation of symphysis, angle, and bilateral condyles)
Day 1 postoperative	No abnormalities in occlusion and jaw movement. No facial nerve deficit
Day 3 postoperative	Discharged in good condition. Antibiotic and analgesic were continued orally. Intermaxillary fixation was done until 2 weeks postoperative
1 week postoperative	Early follow‐up visit
2 weeks postoperative	Follow‐up visit. The intermaxillary fixation was removed
4 weeks postoperative	Follow‐up visit. Mouth opening improved to 40 mm
3 to 6 months postoperative	Postoperative healing was uneventful
3 years postoperative	Final follow‐up—CT scan, clinical photographs showing symmetrical mandibular development, good occlusion, and no growth disturbance

**Figure 5:**
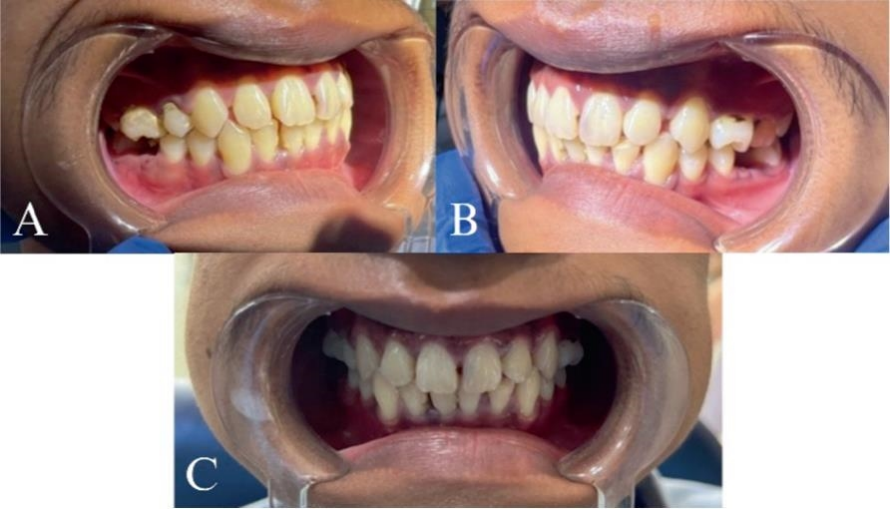
Postoperative occlusion 3 years postoperative. (A) Right lateral view, (B) left lateral view, and (C) frontal view.

Patients were followed up immediately after surgery, first, second, and fourth weeks postoperative. The follow‐up of the patients immediately after the postoperative period was of prime concern to notice the possible changes in the evaluation criteria aforementioned. Postoperatively, mouth opening was 40 mm, and lateral excursions were good (Figure 5). The patients were recalled for further follow‐up at third and sixth month intervals. Postoperative healing was uneventful. A follow‐up panoramic radiograph was taken after a month, and a CT scan was done 3 years after the reconstruction. The patient expressed satisfaction in normal mouth opening and chewing ability. There was no complaint in the cosmetic result, with satisfactory facial symmetry and acceptable scars that did not affect his confidence or daily activities.

**Figure 6:**
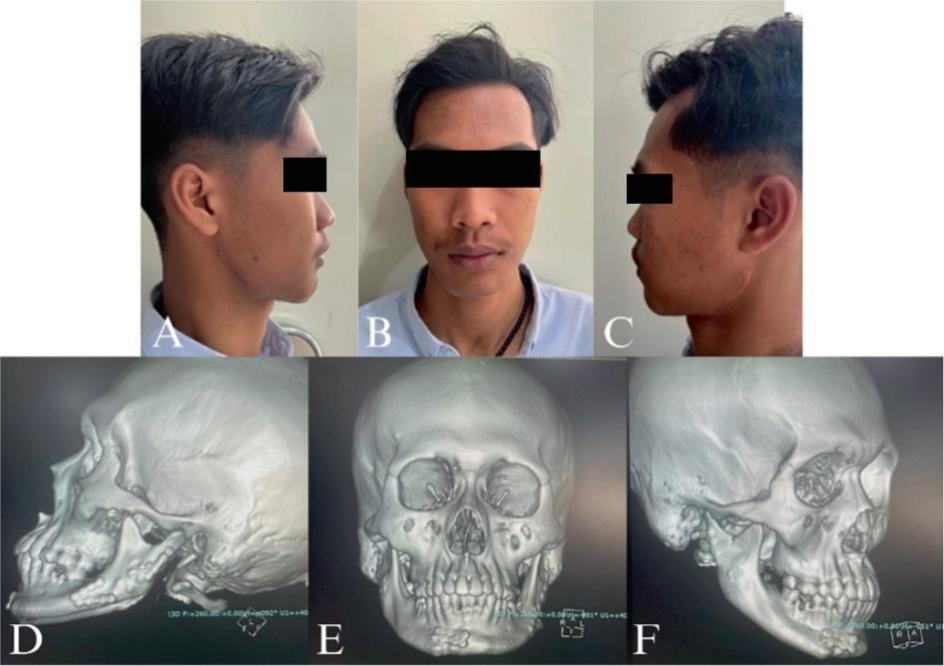
Three‐year follow‐up. Clinical photographs: (A) right lateral view, (B) frontal view, and (C) left lateral view. Head 3D examination: (D) left lateral view, (E) frontal view, and (F) right lateral view.

Postoperative measurement of the ascending mandibular branch revealed a symmetrical development. There were postoperative complications like infection, pain, and functional limitations like restriction of mouth opening or lateral side deviation. The clinical follow‐up was done for 3 years and included measurements of the maximal mouth opening, an analysis of facial symmetry, and vertical height (Figure 6). There was no disturbance of facial growth.

## 4. Discussion

More than half of all facial fractures in children involve the mandible, with the majority occurring at the condylar location [13]. Mandibular condylar fractures in children are a substantial clinical concern as they can lead to long‐term problems if not treated effectively [14]. Pediatric mandibular fractures are associated with several potential complications including facial asymmetry and occlusal discrepancy, generally found in mandibular condylar fractures [15]. Pediatric condylar fractures provide unique concerns for clinicians due to a variety of deforming consequences, particularly ankylosis [5]. Numerous variables, including the direction and site of force application, can produce these fractures [6]. The appropriate treatment of pediatric condylar fractures is critical for reducing the long‐term impact on face growth and development.

Fractures can be broadly classified into two types: intracapsular and extracapsular. However, for diagnostic and practical purposes, they are further separated into three categories: condylar head (intracapsular), condylar neck (extracapsular), and subcondylar fractures. The classifications include undisplaced, deviated, displaced (with medial or lateral override), and dislocated [16]. The therapy of condylar fractures in children is still debated, with clinicians balancing the risks and benefits of conservative versus surgical procedures [17]. Closed treatment of condylar fractures in pediatrics has long been preferred due to concerns about sequelae such as facial nerve injury, external scar, sialocele, and others [14]. Conservative treatment, with or without maxillomandibular fixation and physiotherapy, produced satisfactory to excellent clinical results in the majority of cases [13].

However, the literature indicates that in some circumstances, open reduction and internal fixation may be necessary. The open surgical approach has rapidly become the primary method of addressing these fractures. While conservative management is frequently preferred, open reduction and internal fixation have specific indications in the pediatric population, particularly in cases of bilateral condylar fractures or in older pediatric patients who have reached a more skeletal mature stage and may benefit from the stabilization provided by surgical intervention [18].

The case report presented by Baskara et al. described a comminuted mandibular fracture with significant displacement of the fracture fragments, which constituted one of the indications for the use of open reduction and internal fixation (ORIF) to restore the pretraumatic anatomical relationships [15]. ORIF may be recommended in cases of medial rotation or restricted mouth opening [13, 18]. The ORIF was more effective than conservative treatment for mandibular condyle fractures, with lower rates of asymmetry, residual discomfort, and malocclusion. ORIF of a fracture restores the condylar process to its pretraumatic position, restoring skeletal continuity, normal mandibular position, and normal occlusion [10].

**Figure 7:**
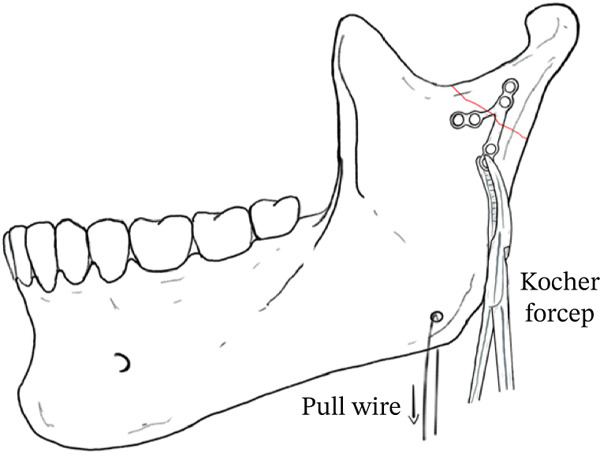
Miniplate fixation with puppet technique.

The “puppet” technique, which aims to restore the correct anatomic relationship and help to achieve a stable fixation, has been proposed as a novel surgical approach that may offer advantages in the pediatric population (Figure 7). With time and mastering of the surgical techniques, the open method has become the preferred choice for condylar fractures. With the developments in instrumentation, surgical techniques, and better understanding of the anatomy, there was a gradual shift in approach toward the treatment of condylar fractures from the nonsurgical/closed method to the surgical/open method^4^.

The condyle also changes with age in children. After active growth, the posterior borders of the ramus and the condyle grow, whereas the anterior border of the condyle undergoes resorption. Lack of pneumatization, fat pockets, and the mixed teeth stage are some of the things that affect this changing process [18]. These all affect how flexible and stable the bone is naturally. When mandibular fractures occur in children, the condyle is often the site of indirect trauma [19].

The condylar bone is generally damaged across the neck after age 5 because it has a thin cortex and a thickened periosteum. Most commonly, medial condylar head dislocation is encountered due to the unopposed pull of the lateral pterygoid. The pediatric condylar unit is highly regenerative and has high osteogenic potential because it has active growth centers and remodeling sites [5]. But the mandible and maxilla reach different stages of skeletal growth in men and women. Girls reach skeletal maturity between the ages of 14 and 16, whereas boys reach maturity between the ages of 16 and 18 [5, 19]. At this age, the body′s ability to make new bones is the same as a child′s, but its ability to change the shape of the condyles decreases.

Bilateral condylar fracture of the mandible is a more disruptive condition in which neither of the condyles retains the normal morphology than to unilateral condylar fracture. Bilateral condylar fractures are common but frequently undertreated. Patients with bilateral condylar fractures may present with an anterior open bite with posterior gagging of occlusion (Kamath). Bilateral condylar fractures lead to loss of Ramal height, accentuated anterior open bite, disruption of articular surfaces, and disc and muscle attachments [6].

In the study conducted by Shende et al., the patient underwent a bilateral neck condylar fracture and utilized a posterior extension of the submandibular incision with open reduction and internal fixation [20]. The treatment was done using effective retraction techniques, including the utilization of the L retractor and traction wire. In contrast, Ragupathy et al. conducted another study in which 16 condylar fracture patients underwent nonsurgical treatment. Within the group that did not undergo surgery, nine patients experienced a decrease in vertical ramus height, whereas six patients had a reduction in mouth openness. The study determined that the condyle is not anatomically normal in radiographs [21].

However, surgical therapy yields more precise clinical and radiographic results. Based on the aforementioned studies, we utilized a modified ORIF with puppet technique for bilateral next condylar fractures in pediatrics patients. In this case, we opted for the surgical approach to assess the clinical and radiological outcomes based on anatomical position, occlusion, nerve, and growth disturbance. The patient has reached the age of 16, which marks the initial stage of maturation of the condylar bones in boys [5]. The condyles undergo mesial displacement, which, in prior trials with nonsurgical methods, led to a reduction in vertical height, growth problems, and other consequences. As previously considered, the open reduction is the most optimal treatment for this case in order to prevent potential long‐term complications.

Open reduction is usually carried out by extra‐oral approaches such as pre‐auricular, submandibular, and retromandibular. In addition to the different skin incision, these techniques may also follow different surgical dissection planes, namely the transparotid and transmasseteric apparoaches. The last one seems to offer more advantages in terms of nerve damage and salivary complications [22]. The classic preauricular approach [23] is well suited to the management of head condylar fractures and very high condylar neck fractures, but can be problematic for treating lower fractures, such as subcondylar described in the patient treated, and is especially troublesome for stabilization. In addition, as reported in the recent literature, this approach is associated with a high risk of nerve injury.

The retromandibular and miniretromandibular [12] approach can be associated with different surgical dissection planes, providing a clear view of the whole ramus by accessing it from the posterior border. In the case of subcondylar fractures, the distance between the skin incision and the area of interest has decreased. The resulting facial scar is located in a relatively inconspicuous area. The recently described “low preauricular approach” maximized these advantages. Since the skin incision coincides with the location of the fracture, the surgeon can avoid working at an oblique angle. The submandibular method is more invasive and has a higher probability of causing damage to the facial nerve, in particular to the marginal mandibulae [22].

In the case presented, miniretromandibular for the condylar treatment provides adequate exposure and reduction of the neck condylar fractured bone [24, 25]. It also minimizes the possibility of a nerve injury and provides good access. In addition, a minimally visible scar appeared compared to a preauricular approach [16]. These methods can restore occlusion, maintain mandibular function, and minimize long‐term problems [13]. We also conduct the modification using the puppet technique to ease the reduction of the fractured fragment by placing a pull wire and manipulating the motion of the bone fragment to achieve the ideal anatomical position.

In this instance, we opt to do prolonged post‐treatment monitoring of the patient to ensure the absence of any growth abnormalities or anatomical complications [26]. During that period of time, both clinical and radiographic examinations were conducted. A CT scan is preferred over an OPG as it has greater accuracy of diagnosis, sensitivity, and specificity [27]. A CT scan section can detect a displaced condylar fracture or a fractured condylar neck. It can reconstruct the condyle in three dimensions, enabling the identification of even the smallest physiological, mechanical, and biological alterations in the TMJ without any overlapping interfering structures.

CT images provide a more precise and comprehensive evaluation of the joint space. The reconstruction method offers a comprehensive anatomical representation and immediately illustrates both static and functional interactions. Additionally, it is beneficial to evaluate crucial factors such as the reduction in vertical ramus height (RH), as this can indicate potential malocclusion, asymmetry, decreased posterior facial height, occlusal cant, and neoarthrosis with the articular eminence [28]. According to this clinical and radiological evidence, it may be concluded that computer‐assisted scans are currently the best imaging techniques for evaluating open reduction and internal fixation [29]. In addition, the systematic review by Sejati et al. explained that the utilization of 3D miniplates might also lower the incidence of complications in mandibular fractures and also strengthen the fracture stability [11].

For the aforementioned condition of bilateral mandibular condyle fractures with medial displacement, it is feasible to use miniretromandibular and submandibular incisions. The challenges of both approaches are associated with accessibility and can potentially be reduced through technical alterations. Fracture fragments with medial displacements can be repositioned using the modification of the ORIF and puppet technique with the help of assistance. In this case, the associated pull wire assists in repositioning the fractured fragments to their correct anatomical alignment. This technique enables the fixation and stabilization of a structure utilizing the titanium miniplate. Upon further examination of the patient, no major complications were found in the growth and development of the mandible, masticatory function, or any other associated issues.

## 5. Conclusions

Thus, it can be concluded that the possibility of ORIF with the puppet technique in pediatric patients with bilateral mandible condylar fractures has demonstrated success and restores early function and anatomic reduction of condylar fractures. Miniretromandibular and submandibular approaches and the puppet technique have demonstrated good results in managing access and providing ease of reduction and fixation. Pediatric condylar fractures require thoughtful consideration in management to not only treat the fractures but also try to avoid future growth complications. It is necessary to follow up on the results over an extended period.

**Nomenclature**
CT: computed tomography
ORIF: open reduction and internal fixation
TMJ: temporomandibular joint

## Author Contributions

Conceptualization: B.P.S.; data curation: R.I.P.; supervision: B.P.S. and Y.B.R.; validation: B.P.S. and Y.B.R.; visualization: R.I.P.; writing – original draft: R.I.P. and Y.B.R.; writing – review and editing: B.P.S. and Y.B.R.

## Funding

No funding was received for this manuscript.

## Disclosure

All authors have read and approved the final version of the manuscript. Bramasto Purbo Sejati as the corresponding author had full access to all of the data in this study and takes complete responsibility for the integrity of the data and the accuracy of the data analysis.

## Ethics Statement

This study is a case report, and our institution does not require an ethical clearance for this type of study. Written informed consent from the patient was acquired for the examination, treatment, and also the publication of this case.

## Conflicts of Interest

The authors declare no conflicts of interest.

## General Statement

*Verification of References.* All references had been confirmed, and none of the cited references have been retracted. The references were managed using Mendeley Reference Manager.

## Data Availability

The authors confirm that the data supporting the findings of this study are available within the article.
